# Social relationships and healthful dietary behaviour: Evidence from over-50s in the EPIC cohort, UK^☆^

**DOI:** 10.1016/j.socscimed.2013.08.018

**Published:** 2014-01

**Authors:** Annalijn I. Conklin, Nita G. Forouhi, Paul Surtees, Kay-Tee Khaw, Nicholas J. Wareham, Pablo Monsivais

**Affiliations:** a Medical Research Council Epidemiology Unit, Institute of Metabolic Science, Cambridge, UK; b UK Clinical Research Collaboration Centre for Diet and Activity Research (CEDAR), University of Cambridge, Cambridge, UK; c Department of Public Health and Primary Care, University of Cambridge, UK

**Keywords:** Social relationships, Social ties, Gender, Interactions, Diet variety, Health behaviour, Chronic disease, UK

## Abstract

Social relationships are an important aspect of a person's social environment that can protect against a wide range of chronic conditions and facilitate recovery from disease. Social relationships have also been linked to dietary behaviour which may be an important pathway through which social circumstances exert their influence on health. Yet, questions remain about which structural aspects of social relationships most affect healthful dietary behaviours and whether different structural components interact to produce a combined effect. Using data from adults (≥50 years) in the European Prospective Investigation of Cancer-Norfolk study (1996–2002), we examined marital status, living arrangement and social isolation in relation to scores for variety of fruit and vegetable intake as a marker of diet quality associated with adverse health outcomes. Data were analysed with multivariable linear regression models for gender-specific and interaction associations. We found that being single or widowed was associated with a lower variety score, particularly vegetable variety, and associations were enhanced when combined with male gender, living alone or infrequent friend contact. Lower variety scores for lone-living were also observed, especially for men. Infrequent friend contact interacted with living arrangement to amplify negative associations of lone-living with variety, with statistically significant differences in contact frequency for vegetable variety. Lower levels of friend contact were associated with reduced variety of fruits and vegetables in a graded trend for both genders; the trend was more pronounced among men. Family contact appeared to have limited association with vegetable variety in men; among women, weekly contact was significantly and positively associated with vegetable variety compared to daily family contact. Results highlight the importance of considering living arrangement and/or frequency of social contact when assessing whether widowed, single or lone-living older adults are at risk of lower fruit and vegetable variety.

## Highlights

• Social relationships affect health and can also influence dietary behaviour.
• We examined joint effects of diverse structural components of relationships on diet.
• We used data from over-50s in an established epidemiological cohort.
• Men fared worse than women in reduced variety from poorer structural social ties.
• Social isolation altered dietary effects of marital status and living arrangement.

## Introduction

Social relationships are known to affect health and survival to an extent comparable with smoking (Holt-Lunstad, Smith, & Layton, 2010). Women and men differ in the number and size of different types of social relationships (Fuhrer, Stansfeld, Chemali, & Shipley, 1999), and in the health impacts (Hessler, Jia, Madsen, & Pazaki, 1995; Kimmel et al., 2000; Shye, Mullooly, Freeborn, & Pope, 1995). Several health-related behaviours are likely to mediate this link for some but not all aspects of a person's social context (Chuang & Chuang, 2008). Diet is a strong candidate for systematic examination along the pathway between social relationships and health so as to better inform chronic disease prevention and promotion of healthy ageing.

A person's social circumstances can influence the type and variety of foods consumed in multiple ways and thereby impact health. Psychosocial mechanisms involved include social support, social influence, social engagement and attachment, and access to resources and material goods (Berkman, Glass, Brissette, & Seeman, 2000). Physiological experiments demonstrated the theory of social facilitation in food intake by showing how the number of people present determines meal size (De Castro & De Castro, 1989) irrespective of time, place, alcohol or snack consumption (De Castro, Brewer, Elmore, & Orozco, 1990). More specifically, more food was consumed when a person ate in the presence of family and friends than when eating around less familiar people such as acquaintances (De Castro, 1994). Others have also found that, regardless of personal taste, a social context in which people eat in the presence of others can influence not only the volume (Wansink & Park, 2001) but also the variety (Pomerlau et al., & The EURO-PREVOB Consortium, 2008) of foods consumed. Among older people, having fewer social contacts or living alone is associated with consuming fewer calories, a less varied diet and fewer portions of fruits and vegetables (Lumbers & Raats, 2006). Food-related behaviours may be particularly influenced by these social factors in widowed men and seniors with limited support (Turrini et al., 2010).

Regarding close relationships, spouses and friends appear to be most concordant in their dietary patterns and, over time, concordances are strongest in spouses (Pachucki, Jacques, & Christakis, 2011). However, spousal or friend influences on dietary behaviours are likely gendered. For example, wives contribute more to husbands' diet quality than the reverse (Schäfer, Schäfer, Dunbar, & Keith, 1999) such that married older men have reported higher intakes of fruits, vegetables and energy-adjusted intakes of antioxidant vitamins and fibre (Tucker, Spiro, & Weiss, 1995). By contrast, friend support appears to contribute more to women's dietary behaviours, particularly when change is needed to make improvements (Barrera, Strycker, MacKinnon, & Toobert, 2008; Kelsey et al., 1996). Similar gender differences are reported in other work on stress and emotions associated with food intake: marital status best predicts stress-related eating in men while lack of emotional support predicts it in women (Laitinen, Ek, & Sovio, 2002). Separate psycho-biological mechanisms involving neuro-endocrine pathways might also link social relationships to dietary behaviours depending on the type (acute or chronic) and perceived severity (threat or challenge) of stress (Adam & Epel, 2007; Willis, Thomas, Garry, & Goodwin, 1987).

Structural and functional components of social relationships likely impact health and diet in different ways (Berkman et al., 2000; Holt-Lunstad et al., 2010). Structural components represent the existence and interconnections of differing social relationships and roles possessed by an individual; this more objective characteristic indicates how relationships are organised and makes support functions possible (Holt-Lunstad et al., 2010; Thoits, 2011). Structural underpinnings of a person's social context are a pathway of influence on diet quality that contains many different types of relationships which remain to be examined for their joint effects (Berkman et al., 2000; Thoits, 2011; Uchino, Bowen, Carlisle, & Birmingham, 2012). We aimed to provide new evidence on synergistic influences on healthful dietary behaviours from multiple social relationships.

This study proposes that structural social relationships comprise unique elements acting independently and synergistically to influence the healthfulness of individual dietary behaviour. We hypothesised that partnership, co-living and frequent social contact would be independently associated with variety of fruits and vegetables, with effects of marital status and living arrangement greater for men and social contact stronger for women. Frequent social contact or co-living was hypothesised to mitigate the negative association of being single or widowed or having rare family contact with variety. Similarly, frequent friend contact would lessen the negative association of living alone with variety.

## Methods

### Study population

We used data collected as part of the EPIC-Norfolk prospective cohort — a component of the European Prospective Investigation of Cancer (EPIC) study in 10 countries (Day et al., 1999). We included 20,274 over-50s from the population-based cohort enrolled in 1993–97 as our interest was adults near the end of working life and beyond to place findings in a healthy ageing context. At entry, over-50s were similar to the total cohort (*n* = 25,639) in terms of health behaviours and other socio-demographic factors. Ethnicity of 99.7% of EPIC-Norfolk participants was of white origin.

Social relationships were assessed in 50–71% of cohort participants using the postal “Health and Life Experiences Questionnaire” (HLEQ) (1996–2000) designed to measure a range of psychosocial variables (Surtees & Wainwright, 2007). The HLEQ instrument has seven sub-sections based on validated questions/scales developed for epidemiological studies with similar objectives, or the EPIC-HLEQ research programme, following standard design principles (Dillman, 1978). Responses to individual questions from over-50s ranged between 10,352 and 14,494. Diet data was provided by 12,292 cohort participants using a food frequency questionnaire (FFQ) during a second clinical assessment (1998–2002). The FFQ was previously validated by comparing to a 16-day weighed food record (Bingham et al., 1994) and nutrient biomarkers (Day, McKeown, Wong, Welch, & Bingham, 2001). Outcome data from over-50s (*n* = 9933) was restricted to FFQ respondents for whom plausible total daily energy (kcal) could be derived (defined as top and bottom 0.5 percentile of energy intake relative to basal metabolic rate). Our available sample therefore included over-50s who responded to social relationship questions, had covariates and follow-up dietary data (*n* = 9580). All volunteers gave written informed consent and the study was approved by the Norwich district ethics committee.

### Structural social relationships

Structural social relationships were studied using three types of connections: marital status, living arrangement, and social isolation. Marital status (*n* = 6257) had five response categories (married/living as married, single, widowed, divorced and separated) and four were used in analyses (partnered, single, widowed, divorced/separated), with partnered (married/living as married) as the reference group. The binary question ‘does anyone live in your household besides you?’ (*n* = 8816) was analysed for living arrangement with co-living as the reference. Social isolation was indicated by the pervasive lack of social contact or communication (including visits, phone calls or letters) with any friend, or with an immediate family not living with a respondent (Holt-Lunstad et al., 2010). Two questions concerned social isolation: participants were asked how often in the past year they had been in contact with any friend (friend contact, *n* = 8442), and with immediate family not living with them (family contact, *n* = 8388). Both questions had seven response categories (daily, several time/week, about once/week, 2–3 times/month, about once/month, less than once/month, never or hardly ever) which were combined into four categories for analyses (daily, weekly, monthly, never/rare) with daily contact as reference.

### Healthful eating behaviours

Participants reported their average consumption of a pre-specified number of fruit (*n* = 11) and vegetable (*n* = 26) products over the last year, with nine standard response categories (from never/less than once per month to six or more/day) (Willett, 2013). Average daily consumption of fruits and vegetables (g/day) was calculated from self-reported frequencies using an established method (Bingham et al., 1994). We used daily consumption of each unique fruit or vegetable product to derive a score for fruit variety and vegetable variety (no. items/day). The score summed the total number of unique products reported to be consumed (>0 g/day), irrespective of total quantity. The variety score was used as a proxy for healthfulness of the diet since similar variety scores have been associated with reduced risk of type 2 diabetes in this cohort (Cooper et al., 2012). Other studies have demonstrated the reproducibility and validity of variety scores for nutritional adequacy in older populations (Bernstein et al., 2002; Drewnowski, Renderson, Driscoll, & Rolls, 1997).

### Socio-demographic variables

Concurrent socio-demographic variables included: self-reported general health status (excellent, good, moderate, poor) and smoking status (current, former, never); and, clinically measured BMI. Some variables were assessed at entry but are generally time-invariant: education (no qualification, O-level, A-level, degree), date of birth (continuous age), gender (male, female) and social class (professional, managerial and technical, skilled non-manual, skilled manual, partly skilled, unskilled).

### Data analysis

Descriptive statistics summarised socio-demographic characteristics and crude mean variety scores in relation to social exposure variables. Multivariable linear regression models assessed cross-sectional associations between each social relationship and the fruit or vegetable variety score. Our *a priori* strategy for main analyses was to examine (1) gender-stratified associations, and (2) interactions among different social relationships. We aimed to investigate whether overall associations of, for example, different categories of marital status differed when a second structural measure was considered (e.g. friend contact, living arrangement). Interaction terms therefore included: marital status by living arrangement and by friend contact; family contact by living arrangement and by friend contact; and living arrangement by friend contact. Friend contact was dichotomised into ‘frequent’ (daily, weekly, and several times a month) and ‘infrequent’ (about once a month, less than once a month and never/hardly ever) for interaction analyses. We separately tested significance of gender differences (*p* < 0.05) using a sex interaction term. All analyses adjusted for total energy intake (Kcal), age, education and (as appropriate) gender. As known confounders, each is associated with the exposure and independently with diet (Davis, Murphy, Neuhaus, Gee, & Quiroga, 2000; Donkin et al., 1998; Irz et al., 2013).

Sensitivity analyses additionally adjusted for quantity of fruit (for fruit variety) and vegetable (for vegetable variety) since higher variety is associated with increased quantity of these foods and with more energy intake (Büchner et al., 2010). We also controlled for poor health as a potential confounder of daily family contact in women by adding pre-existing self-reported health status, or cancer, stroke and high blood pressure, to relevant models. Separate models also controlled for social class to determine whether observed estimates changed.

All statistical analyses were conducted using Stata 12.1 (StataCorp, 2011). Findings were based on beta-coefficients and 95 percent confidence intervals; significance for interaction analyses was set at *p* < 0.10.

## Results

Our sample's average age was 62 years, with 55 percent female. A majority (83%) reported being in excellent/good general health and 51% were ever smokers. Over half were educated to degree/A-level, although more men (62%) than women (48%) had higher education. Those in the top-two social classes comprised 46 percent of the sample, and more women (4%) than men (2%) had unskilled occupations. Both men and women were generally overweight at follow-up, with mean BMI of 26.8 and 26.7 respectively. Women, however, reported consuming fewer total calories than men (1850 versus 2087 kcal/day). Variety scores were normally distributed and crude means for fruit variety and vegetable variety were higher in women than men (7.7 and 16.5 versus 6.7 and 15.9, respectively). Few over-50s reported no average daily consumption of fruits (*n* = 55) or vegetables (*n* = 6) and therefore scored zero.

Socio-demographic characteristics were generally evenly distributed across categories of each social relationship measure (Table 1). There were higher proportions of women in non-partnered, lone-living, and daily contact categories. A higher proportion of ever smokers were found at greater levels of social isolation (from friend and family). Lower levels of friend contact had decreasing proportions of higher educated participants, whereas decreasing family contact was associated with increasing proportions of higher education.

### Gender-specific associations

Men and women differed in associations between marital status and both dietary outcomes (Supplementary Table 1). The negative dietary associations with all non-partnered categories were stronger in men. For example, compared to partnered men, widowed men had a −2.17 unit difference (*p* < 0.001) in vegetable variety which was significantly different (*p* = 0.005) from widowed women who had a −0.79 unit difference (*p* < 0.001) in score compared to partnered women. Single men and women differed significantly (*p* = 0.018) in lower vegetable variety scores compared to partnered counterparts. Negative associations between lone-living and variety scores were also stronger in men: lone-living men had a −1.46 unit difference (*p* < 0.001) in vegetable variety score which was significantly different (*p* = 0.001) from the −0.66 unit difference (*p* < 0.001) in score for lone-living women, compared to co-living counterparts.

Women and men with decreasing friend contact had lower variety scores. Notably, associations with fruit variety appeared more smoothly graded in women and slightly more pronounced in men having rare/no contact. The relationship between family contact and variety scores was less consistently patterned. Lower frequencies of family contact were associated with lower fruit variety scores and rare/no contact was similarly negative for both genders. By contrast, decreasing family contact seemed to have limited association with vegetable variety in men whereas weekly contact had a 0.56 unit difference (*p* = 0.001) in score in women compared with daily family contact. Given this unexpected direction, we further adjusted for prior chronic physical conditions in case daily family contact was due to women's poor health. Consistent with other studies (Kaplan et al., 1988), additional correction minimally attenuated observed estimates and did not substantially alter results. Inclusion of fruit quantity (for fruit variety) or vegetable quantity (for vegetable variety) also did not change reported associations. Sensitivity analyses that included additional adjustment for social class resulted in no material difference in associations (data not shown).

### Marital status and healthful dietary behaviours, by living arrangement or friend contact

**Fig. 1:**
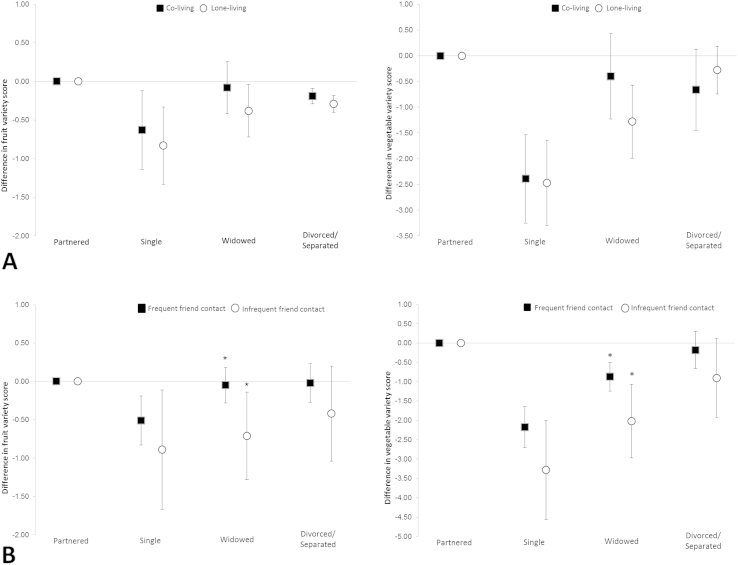
Association between marital status and variety of fruits or vegetables by living arrangement (A) and by friend contact (B). *Significant interaction by friend contact in widowed (fruit variety, *p* = 0.034; vegetable variety, *p* = 0.026).

Fig. 1 and Supplementary Table 2 show how associations between marital status and variety differ by living arrangement or friend contact. Lone-living single and widowed over-50s had, respectively, a −0.83 (*p* = 0.001) and −0.38 (*p* < 0.05) unit difference in fruit variety scores, which were slightly lower than unit differences for those in shared accommodation (single: −0.63, *p* < 0.05; widowed: −0.08, *p* > 0.05), compared to partnered counterparts (Fig. 1A). Differences in living arrangement, however, were non-significant. For vegetable variety, we found widowed over-50s living alone had a −1.28 unit difference (*p* < 0.001) compared to partnered counterparts whereas the association for co-living over-50s was limited (Beta = −0.40, *p* > 0.05); again living arrangement differences were non-significant. For divorced/separated over-50s, differences in variety scores between co- and lone-living were reversed (Fig. 1A): those living alone had a −0.28 unit difference (*p* > 0.05) in vegetable variety versus a −0.66 unit difference (*p* > 0.05) for those co-living (compared to partnered counterparts).

Fig. 1B shows the generally negative association between non-partnered categories and fruit or vegetable variety was larger when friend contact was infrequent. Widowed over-50s with infrequent friend contact showed a significantly (*p* = 0.034) lower unit difference of −0.71 (*p* < 0.05) in fruit variety scores than the −0.05 unit difference (*p* > 0.05) for those with frequent contact (compared to partnered counterparts). Similarly, unit differences in vegetable variety scores were significantly lower (*p* = 0.026) for widowed over-50s with infrequent friend contact than for those with frequent contact (−2.02 versus −0.87; both *p* < 0.001) compared to partnered counterparts—a difference of 1.15 items/day. Non-significant differences in friend contact were also observed in variety scores of single over-50s (Fig. 1B).

### Family contact and healthful dietary behaviours, by living arrangement or friend contact

**Fig. 2:**
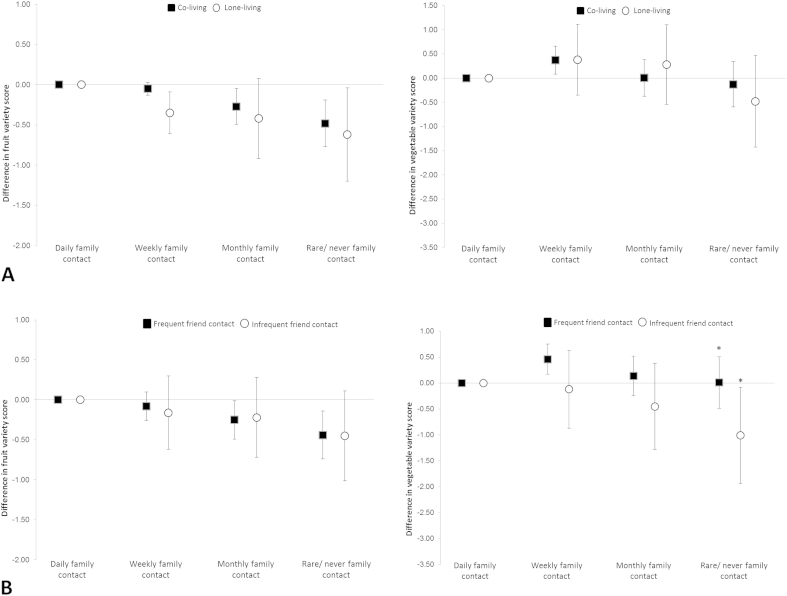
Association between family contact and variety of fruits or vegetables by living arrangement (A) and by friend contact (B). *Significant interaction by friend contact in rare/no family contact (*p* = 0.056).

Family contact and variety scores showed differences by living arrangement and friend contact (Fig. 2 and Supplementary Table 3). Fig. 2A shows fruit variety decreased as family contact decreased when over-50s lived alone. Compared with daily family contact, lone-dwellers with rare/no family contact had a −0.62 unit difference (*p* < 0.05) in score and those in shared accommodation had a −0.48 unit difference (*p* = 0.001). Decreasing family contact had limited association with vegetable variety for co- and lone-living over-50s, apart from weekly contact (versus daily) appearing protective in co-living over-50s. Differences by friend contact frequency were observed only for associations of family contact with vegetable variety (Fig. 2B). Weekly and monthly family contact among over-50s with frequent friend contact showed, respectively, a 0.46 (*p* < 0.05) and 0.14 unit difference in vegetable variety but rare/no contact did not (reference daily contact). By contrast, decreasing family contact was associated with lower scores among over-50s with infrequent friend contact such that rare/no contact showed a −1.01 unit difference (*p* < 0.05) compared to daily contact. Differences in friend contact of one vegetable item/day were significant (*p* = 0.056) for adults with rare/no family contact.

### Living arrangement and healthful dietary behaviours, by friend contact

**Fig. 3:**
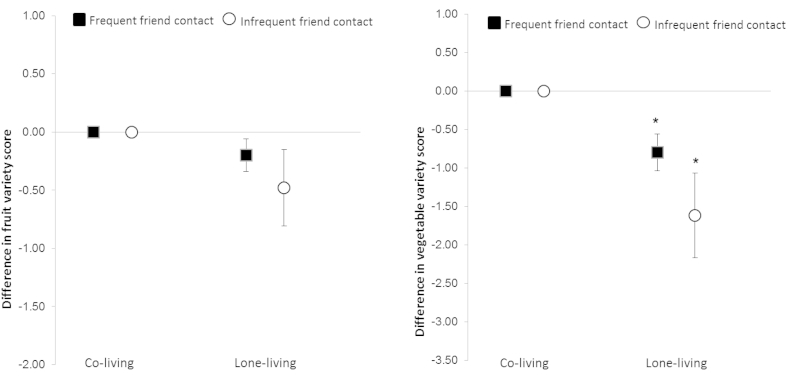
Association between living arrangement and variety of fruits or vegetables by friend contact. *Significant interaction by friend contact in lone-living adults (*p* = 0.007).

A significant negative association of lone-living with both scores appeared amplified when friend contact was infrequent (Fig. 3 and Supplementary Table 4). Compared with co-living, lone-dwellers with infrequent friend contact had a −0.48 unit difference (*p* < 0.05) in fruit variety which was not significantly different from the −0.20 unit difference (*p* < 0.05) for lone-dwellers with frequent friend contact. The association of lone-living with vegetable variety revealed significant differences (*p* = 0.007) between infrequent and frequent friend contact (−1.62 versus −0.80; both *p* < 0.001), representing 0.82 different vegetable items/day.

## Discussion

The association of social relationships with diet quality is well characterised in the literature but less is known about combined influences of structured social experiences. Our findings demonstrated that men fared worse than women in the negative associations of non-partnered, lone-living and rare/no friend contact with variety of fruits or vegetables. Associations between family contact and variety by gender were less clear. Our observation that over-50s who are widowed, lone-living or had rare/no family contact reported consuming, respectively, 1.15, 0.82 and 1.00 fewer different vegetable products daily when friend contact was infrequent versus frequent is clinically meaningful. We previously showed in this cohort that consuming three additional vegetable items per week lowered diabetes risk by 13%, independent of quantity and other potential confounders, and that the inverse association with diabetes was linear within the normal range of variety of intake (5.5–11.4 items/week) (Cooper et al., 2012). Across 10 EPIC study countries, increasing variety in vegetable and/or fruit consumption reduced risk of certain cancers (Büchner et al., 2010; Jeurnink et al., 2012). Many national and international bodies recommend eating a variety of fruits and vegetables without specifying targets for adequate/optimal variety (USDA & DHHS, 1980; WHO/FAO, 2003). Nevertheless, this cohort compares with mean vegetable variety in US community-dwelling elderly (11 items/week) (Marshall, Stumbo, Warren, & Xie, 2001). Our results have public health implications for supporting healthy ageing since over-50s are more likely to experience transitions in the structure of their social relationships, moving from multiple to more limited or no ties.

### Gender-specific findings and potential mechanisms in the context of previous studies

Overall, our results support gender differences in the roles of marital status, living arrangement and social isolation, in healthful dietary behaviours. Results confirm our hypotheses that marital status and living arrangement influences on fruit or vegetable variety were greater for men. Findings were mixed for the relationship between social isolation and variety. Consistent with previous research (Hessler et al., 1995), women had more frequent social contact than men in the sample. Although the associations of friend and family contact were stronger in men for fruit variety, we did find clearer patterning in women. Thus, findings did not concur with pre-specified hypotheses and may be explained by the fact that social isolation, defined by limited *structures*, affects men more whereas women may be more influenced by *functional* aspects, including emotional support (Stringhini et al., 2012). Results for family contact and vegetable variety were most surprising: weekly contact (versus daily) was significantly positively associated in women, but men showed limited associations.

Previous research supports the finding that isolation from friends or family, lone-living and no intimate partnership are each associated with diets limited in variety and/or low in nutritional quality (Dean, Raats, Grunert, Lumbers, & The Food in Later Life Team, 2009; Lilley, 1996; Wenrich, Brown, Miller-Day, Kelley, & Lengerich, 2010). Other US or UK studies indicate that older men living or eating alone are at greater risk of poor diets (Davis, Murphy, Neuhaus, & Lein, 1990; Holmes, Roberts, & Nelson, 2008), with living arrangement influencing fruit and vegetable consumption to significant levels in older British men but not women (Donkin et al., 1998). One reason living or eating alone might reduce fruit or vegetable variety is there are no economies of scale in food procurement and preparation. A Swedish study of recently bereaved older women found they perceived the financial constraints associated with lone-living to affect their management of food shopping and cooking (Sidenvall, Nydahl, & Fjellström, 2001). Since older single women are typically more disadvantaged financially than men (Lyon & Colquhoun, 1999), we might have expected stronger negative associations for women but this was not observed.

Our results might be explained instead by lack of motivation to prepare a meal when living/eating alone since the psycho-social mechanism of social engagement is absent (Berkman et al., 2000). A substantial body of research indicates people derive social meaning from cooking for others and from sharing a meal, representing both a social and a food event (Lumbers & Raats, 2006). Lack of motivation to cook due to the effort involved would more likely affect the consumption of vegetables which generally require more preparation than fruits. This is consistent with our finding of a greater negative association with the variety score for vegetables than for fruits among single and widowed versus partnered over-50s.

Poor motivation might further explain the greater negative associations seen in men. Men may be less motivated to prepare a meal when living alone because they are less equipped than women regarding cooking skills and being self-reliant while in a partnership (Bennett, Hughes, & Smith, 2003; Lumbers & Raats, 2006). A study comparing single and married elderly men and women found that single elderly women (87% widows) made food decisions independent of others and had better quality diets than the other groups (Schäfer & Keith, 1982). Lack of motivation to eat a variety of vegetables or fruits is also more likely among men who commonly perceive cooking as burdensome particularly when widowhood demands they adopt new food-related tasks and consequent social roles, rather than as freedom which women can experience in widowhood (Davidson, 2001). Finally, personal motivation has been reported as the main influence on men aiming to improve dietary behaviours, rather than socio-ecological resources (Barrera et al., 2008; Kelsey et al., 1996).

### Combined influence of social relationships

Combinations of different social relationships were notable in differentially influencing on healthful dietary behaviours of over-50s. We saw synergy of action between marital status and living arrangement, and between marital status and friend contact, thus confirming hypotheses that negative associations of being single or widowed with variety may be mitigated by shared accommodation or frequent social contact. Results clearly indicated that friend contact played a significant role in the extent to which being widowed showed reduced variety, suggesting that widowed persons at risk of consuming fewer different fruit or vegetable products are those with infrequent contact. Equally, infrequent friend contact amplified the extent of reduced variety among older lone-dwellers. In addition, over-50s having rare/no family contact ate fewer different vegetables when friend contact was infrequent but not when contact was frequent.

Our results suggested further that co-living might mitigate the potentially negative association of being widowed with variety, particularly of vegetables. Others have also reported that living alone, versus co-living, limited the variety of vegetables and fruits (and number of meals) eaten by single older adults compared to married counterparts (Donkin et al., 1998; Shatenstein, Nadon, & Ferland, 2004). One interpretation for these interaction results might be the buffering role that occurs from companionship availability separate from social engagement provided through intimate partnership. Physiological studies of elderly suggest the existence of a confidant relationship can mitigate the general response to stressful stimuli (Willis et al., 1987) and also stress-related loss of appetite (McIntosh, Shifflett, & Picou, 1989).

The role of living arrangement in healthful dietary behaviours was also modified by friend contact, supporting the hypothesis that frequent friend contact would lessen the negative association of lone-living with variety. The combination of lone-living and infrequent friend contact was notably worse than the combination of lone-living and frequent friend contact for reducing intake of different fruit and especially vegetable products. Our results concur with previous work indicating reduced variety of healthful foods may be caused not solely by living alone but by loneliness, since frequent friend contact provides the opportunity for social interaction at mealtimes which is known to improve the diets of lone-living elderly (Lumbers & Raats, 2006).

It is also possible that friend contact modified associations through functional support. Stronger effects of functional components have been found over structural measures in predicting mental health (Fuhrer et al., 1999) or diet quality (Nicklett et al., 2012). Yet, others have not found support functions explained the independent associations between social isolation and higher mortality risk (Steptoe, Shankar, Demakakos, & Wardle, 2013). It is argued that participation in social relationships itself results in health behaviours because of the opportunities for sociability, meaningful roles and shared norms which do not result from social support per se (Berkman et al., 2000). Since structural and functional components might activate similar psychological mechanisms (Thoits, 2011), future research should examine mediation of functional components in associations between relationship structures and diet quality, using behaviour-specific measures of perceived social support which are more predictive than generic indicators (Sallis, Grossman, Pinski, Patterson, & Nader, 1987).

### Strengths and weaknesses

Exposures and outcomes were self-reported and like all such variables may be subject to recall or social desirability bias. For example, older ages or lack of food preparation involvement may reduce recall of the full range of products consumed. Social desirability favouring variety may be associated with more social ties and falsely increased outcomes. Lower education and social class of widowed over-50s, or stress from recent bereavement, may affect reported variety. Errors in diet recall ability have previously been shown to be greater in relation to education and income but not age (Kuzma & Lindsted, 1990). Adjustment for education and social class in sensitivity analyses will mitigate any effects of bias in outcome ascertainment on observed estimates. Poor diet recall may be less than expected as the FFQ may perform better in older adults with set food repertoires and meal routines than younger cohorts with dynamic eating patterns.

It is important to investigate self-reported relationships which measure perceived food-related resources since perceptions might impact variety more than actual resources (Dean et al., 2009). Future work could examine measures not included here such as other types of relationship structures (e.g. social networks, social integration), functional components, quality/satisfaction of a relationship, or quantity (e.g. number of friends). Moreover, social relationship measures were assessed as a state but different relationship transitions can change dietary behaviour in opposite directions. For example, becoming divorced or widowed may decrease vegetable intake, compared to remaining married; while men and women who remarry may increase vegetable consumption compared to unmarried counterparts (Eng, Kawachi, Fitzmaurice, & Rimm, 2005; Lee et al., 2005). Thus there may have been misclassification of exposures stemming from changes to participants' relationship category/level in the interval between the questionnaire assessment of social relationships and subsequent diet. Such misclassification would be non-differential since it was unlikely to have been related to the outcomes examined and hence would bias results towards the null.

Our findings are subject to residual confounding from income which was not collected in the cohort and might result in observed associations being larger than true associations. Although low income can be a barrier to consuming fruits and vegetables, income is not consistently associated with elderly consumption and has not been found to explain associations between living arrangement and diet (Bihan et al., 2010; Payette & Shatenstein, 2005; Rosenbloom & Whittington, 1993). By contrast, education is consistently associated with elderly diet quality and shows stronger gradients than income (Irz et al., 2013). As our sample differed from the full cohort only in greater prevalence of higher education and social class, findings cannot be generalised to lower SES populations. More work is also needed on non-white or younger groups and might examine other dietary components or patterns.

Our study has several strengths: a large sample size, gender analyses, effect modification, multiple known confounders, and four separate measures of three relationship types. The particular strength of our work is in considering interactions of social relationships in relation to diet. In doing so, we begin to capture the complex reality of an individual's heterogeneous life circumstances wherein multiple roles and shared norms interact and mutually reinforce to produce unique social environments (Verloo, 2006). Future research must continue to examine how structural aspects of social relationships connect with each other to produce a combined effect across different configurations of an older person's lived experience to influence healthful dietary behaviours, as called for by public health and policy researchers (Killoran & Kelly, 2010).

A further strength is our use of variety scores with unique attributes: they are a good marker of diet quality (Bernstein et al., 2002; Drewnowski et al., 1997; Krebs-Smith, Smiciklas-Wright, Guthrie, & Krebs-Smith, 1987); have shown utility for chronic disease aetiology (Büchner et al., 2010; Cooper et al., 2012; Jeurnink et al., 2012); and variety of fruits and vegetables is long recommended as critical to healthful eating (DNHW, 1982; USDA & DHHS, 1980). Finally, apart from fewer smokers and minimal ethnic minorities, this cohort has similar characteristics to the general UK population (Day et al., 1999). Thus, findings from our sample could be generalised to other white European-origin higher socioeconomic status over-50s.

## Conclusion

This study confirms the gender-specific associations of social relationships with variety of intake of fruits and vegetables in a UK population, and contributes new evidence on the combined influence of structural components of relationships. Variety scores of men were more influenced than those of women by marital status, living arrangement or friend contact. Thus, structural interventions aimed at increasing availability of social relationships by reducing social isolation or supporting recent widows/widowers are likely important for promoting healthful dietary behaviours, particularly among men. Results also highlight the importance of considering living arrangement and friend contact when assessing whether widowed or single over-50s are at risk of eating fewer different fruits or vegetables. The influence of frequent friend contact in combination with either lone-living or rare/no family contact should also be considered for supporting healthful eating among older people. Future research needs to analyse potential mediation of functional components in the association between structured social experiences and diet quality. Further examination of men's and women's physiological responses to the type and quality of social relationships will also be useful to inform psycho-biological mechanisms of social life influences on healthful dietary behaviours.

## Figures and Tables

**Table 1:** Characteristics of structural social relationships for over-50s in EPIC-Norfolk.

	Women	Mean age	A-level/degree educated	Social classes I & II^a^	Poor/moderate health	Ever smoker	Mean (SD) BMI	Mean (SD) fruit variety score^b^ (0–11)	Mean (SD) vegetable variety score^b^ (0–26)
*Marital status (n* *=* *6257)*									
Partnered (*n* = 5040)	52%	62	56%	47%	15%	50%	26.7 (3.8)	7.3 (2.4)	16.4 (3.9)
Single (*n* = 270)	62%	62	59%	44%	19%	45%	26.7 (4.8)	6.9 (2.6)	14.3 (4.6)
Widowed (*n* = 597)	84%	67	45%	33%	22%	50%	26.8 (4.4)	7.4 (2.4)	15.1 (4.3)
Divorced/separated (*n* = 350)	73%	60	62%	44%	21%	52%	26.6 (4.5)	7.4 (2.6)	16.4 (4.1)
*Living arrangement (n* *=* *8816)*									
Shared (*n* = 7243)	52%	61	55%	48%	16%	52%	26.7 (3.8)	7.3 (2.4)	16.4 (3.9)
Alone (*n* = 1573)	71%	65	53%	41%	19%	50%	26.8 (4.3)	7.2 (2.5)	15.4 (4.3)
*Friend contact (n* *=* *8442)*									
Daily (*n* = 431)	68%	63	61%	48%	16%	49%	27.6 (4.2)	7.8 (2.4)	16.7 (4.1)
Weekly (*n* = 5277)	58%	62	56%	48%	15%	49%	26.7 (3.9)	7.4 (2.4)	16.5 (3.9)
Monthly (*n* = 2005)	53%	62	54%	46%	18%	52%	26.7 (3.9)	7.2 (2.4)	16.2 (3.9)
Rare/never (*n* = 729)	41%	62	52%	42%	19%	58%	26.8 (3.9)	6.5 (2.6)	15.2 (4.3)
*Family contact (n* *=* *8388)*									
Daily (*n* = 875)	65%	61	45%	43%	19%	48%	27.3 (4.0)	7.5 (2.3)	16.1 (4.0)
Weekly (*n* = 5849)	57%	62	56%	48%	15%	50%	26.7 (3.9)	7.4 (2.4)	16.5 (4.0)
Monthly (*n* = 1148)	47%	63	58%	47%	17%	55%	26.7 (3.8)	7.0 (2.5)	16.0 (4.1)
Rare/never (*n* = 516)	40%	63	57%	44%	21%	56%	26.9 (3.6)	6.7 (2.5)	15.6 (4.1)

Note: ^a^ Social class I = professional; class II = managerial and technical occupations. ^b^ Higher variety score is associated with better health outcomes, e.g. reduced type 2 diabetes risk; hence, serving as a proxy for healthful dietary behaviours. Measurement time-points were: gender, age, education, class (1993–1997); marital status, living arrangement, friend contact and family contact (1996–2000); diet, health, BMI and smoking status (1998–2002).
