# Supplementary material for: Social relationships and healthful dietary behaviour: Evidence from over-50s in the EPIC cohort, UK
Source: Soc Sci Med. 2014 Jan;100(100):167–75. doi: 10.1016/j.socscimed.2013.08.018 (PMC3969105; doi:10.1016/j.socscimed.2013.08.018)
Supplement: Supplementary file 1 [file mmc1.doc]

**Supplementary Table 1. Association between structural social relationships and variety of fruits or vegetables by gender**

|  | **Fruit variety** | |  | **Vegetable variety** | |
| --- | --- | --- | --- | --- | --- |
|  | **Women** | **Men** |  | **Women** | **Men** |
| ***Marital Status*** |  |  |  |  |  |
| **Partnered** | ̶ | ̶ |  | ̶ | ̶ |
| **Single** | -0.44 (-0.78, -0.09) | -0.80 (-1.29, -0.32) |  | -1.85 (-2.44, -1.25)^a^ | -3.01 (-3.77, -2.25)^a^ |
| **Widowed** | -0.05 (-0.27, 0.17) | -0.49 (-0.99, 0.02) |  | -0.79 (-1.18, -0.41)^b^ | -2.17 (-2.96, -1.37)^b^ |
| **Divorced/ Separated** | 0.02 (-0.27, 0.30) | -0.44 (-0.94, 0.06) |  | -0.21 (-0.70, 0.28) | -0.65 (-1.44, 0.13) |
| ***Living arrangement*** |  |  |  |  |  |
| **Shared** | ̶ | ̶ |  | ̶ | ̶ |
| **Alone** | -0.18 (-0.33, -0.03) | -0.34 (-0.58, -0.09) |  | -0.66 (-0.92, -0.39)^c^ | -1.46 (-1.84, -1.08)^c^ |
| ***Social isolation – friend contact*** |  |  |  |  |  |
| **Daily** | ̶ | ̶ |  | ̶ | ̶ |
| **Weekly** | -0.12 (-0.38, 0.14) | -0.32 (-0.75, 0.10) |  | -0.11 (-0.56, 0.35) | -0.02 (-0.68, 0.65) |
| **Monthly** | -0.35 (-0.64, -0.07) | -0.37 (-0.81, 0.07) |  | -0.33 (-0.82, 0.16) | -0.26 (-0.95, 0.43) |
| **Rare/ no** | -0.76 (-1.11, -0.41) | -1.00 (-1.47, -0.52) |  | -1.12 (-1.73, -0.50) | -1.23 (-1.98, -0.49) |
| ***Social isolation – family contact*** |  |  |  |  |  |
| **Daily** | ̶ | ̶ |  | ̶ | ̶ |
| **Weekly** | 0.01 (-0.18, 0.21) | -0.27 (-0.56, 0.03) |  | 0.56 (0.23, 0.90) | 0.01 (-0.45, 0.47) |
| **Monthly** | -0.17 (-0.43, 0.09) | -0.48 (-0.82, -0.14) |  | 0.05 (-0.40, 0.50) | -0.22 (-0.76, 0.31) |
| **Rare/ no** | -0.58 (-0.93, -0.23) | -0.53 (-0.92, -0.13) |  | -0.60 (-1.21, 0.01) | -0.30 (-0.92, 0.31) |

Differences by gender in variety score are illustrated as beta-coefficients and 95% CI adjusted for total energy intake, age and education. Numbers analysed were gender-specific for marital status (Women: n=3,523; Men: n=2,729), living arrangement (Women: n=4,892; Men: n=3,918), contact with any friend (Women: n=4,729; Men: n=3,708), and contact with immediate family not living with participant (Women: n=4,661; Men: n=3,721). Significant interaction by gender: ^a^ single: p=0.018; ^b^ widowed: p=0.005; ^c^ alone: p=0.001).

**Supplementary Table 2. Association between marital status and variety of fruits or vegetables by living arrangement and by friend contact**

|  | **Fruit variety** | |  | **Vegetable variety** | |
| --- | --- | --- | --- | --- | --- |
|  | **Co-living** | **Lone-living** |  | **Co-living** | **Lone-living** |
| ***Marital Status*** |  |  |  |  |  |
| **Partnered** | ̶ | ̶ |  | ̶ | ̶ |
| **Single** | -0.63 (-1.15, -0.12) | -0.83 (-1.33, -0.33) |  | -2.39 (-3.24, -1.53) | -2.47 (-3.30, -1.64) |
| **Widowed** | -0.08 (-0.59, 0.42) | -0.38 (-0.81, 0.04) |  | -0.40 (-1.23, 0.44) | -1.28 (-1.98, -0.57) |
| **Divorced/ Separated** | -0.19 (-0.67, 0.29) | -0.29 (-0.76, 0.18) |  | -0.66 (-1.46, 0.13) | -0.28 (-1.06, 0.50) |
|  |  |  |  |  |  |
|  | **Fruit variety** | |  | **Vegetable variety** | |
|  | **Frequent friend contact** | **Infrequent friend contact** |  | **Frequent friend contact** | **Infrequent friend contact** |
| ***Marital Status*** |  |  |  |  |  |
| **Partnered** | ̶ | ̶ |  | ̶ | ̶ |
| **Single** | -0.51 (-0.83, -0.19) | -0.89 (-1.66, -0.11) |  | -2.17 (-2.70, -1.64) | -3.28 (-4.57, -2.00) |
| **Widowed** | -0.05 (-0.28, 0.18)^a^ | -0.71 (-1.28, -0.14)^a^ |  | -0.87 (-1.25, -0.50)^b^ | -2.02 (-2.96, -1.07)^b^ |
| **Divorced/ Separated** | -0.02 (-0.30, 0.27) | -0.42 (-1.03, 0.20) |  | -0.18 (-0.65, 0.30) | -0.90 (-1.92, 0.12) |

Differences by living arrangement (n= 5875) and by friend contact (n=5636) in variety score are illustrated as beta-coefficients and 95% CI adjusted for total energy intake, age, gender and education. Significant interaction by friend contact in widowed over-50s: ^a^ fruit variety (p=0.034); ^b^ vegetable variety (p=0.026).

**Supplementary Table 3. Association between family contact and variety of fruits or vegetables by living arrangement and by friend contact**

|  | **Fruit variety** | |  | **Vegetable variety** | |
| --- | --- | --- | --- | --- | --- |
|  | **Co-living** | **Lone-living** |  | **Co-living** | **Lone-living** |
| ***Family contact*** |  |  |  |  |  |
| **Daily** | ̶ | ̶ |  | ̶ | ̶ |
| **Weekly** | -0.05 (-0.23, 0.13) | -0.35 (-0.79, 0.09) |  | 0.37 (0.07, 0.66) | 0.38 (-0.34, 1.11) |
| **Monthly** | -0.27 (-0.50, -0.05) | -0.42 (-0.92, 0.08) |  | 0.002 (-0.37, 0.38) | 0.28 (-0.54, 1.10) |
| **Rare/No** | -0.48 (-0.76, -0.19) | -0.62 (-1.20, -0.04) |  | -0.13 (-0.60, 0.34) | -0.48 (-1.43, 0.47) |
|  |  |  |  |  |  |
|  | **Fruit variety** | |  | **Vegetable variety** | |
|  | **Frequent friend contact** | **Infrequent friend contact** |  | **Frequent friend contact** | **Infrequent friend contact** |
| ***Family contact*** |  |  |  |  |  |
| **Daily** | ̶ | ̶ |  | ̶ | ̶ |
| **Weekly** | -0.08 (-0.25, 0.10) | -0.16 (-0.61, 0.30) |  | 0.46 (0.16, 0.75) | -0.12 (-0.87, 0.63) |
| **Monthly** | -0.25 (-0.48, -0.01) | -0.22 (-0.72, 0.28) |  | 0.14 (-0.24, 0.52) | -0.45 (-1.28, 0.38) |
| **Rare/No** | -0.44 (-0.74, -0.14) | -0.45 (-1.01, 0.11) |  | 0.01 (-0.49, 0.51)^a^ | -1.01 (-1.93, -0.08)^a^ |

Differences by living arrangement (n=8344) and by friend contact (n=8047) in variety score are illustrated as beta-coefficients and 95% CI adjusted for total energy intake, age, gender and education. ^a^ significant interaction by friend contact in over-50s with rare/no family contact (p=0.056).

**Supplementary Table 4. Association between living arrangement and variety of fruits or vegetables by friend contact**

|  | **Fruit variety** | |  | **Vegetable variety** | |
| --- | --- | --- | --- | --- | --- |
|  | **Frequent friend contact** | **Infrequent friend contact** |  | **Frequent friend contact** | **Infrequent friend contact** |
| ***Living arrangement*** |  |  |  |  |  |
| **Shared** | ̶ | ̶ |  | ̶ | ̶ |
| **Alone** | -0.20 (-0.35, -0.06) | -0.48 (-0.81, -0.15) |  | -0.80 (-1.04, -0.56)^a^ | -1.62 (-2.17, -1.07)^a^ |

Differences by friend contact (n=8403) in variety score are illustrated as beta-coefficients and 95% CI adjusted for total energy intake, age, gender and education. ^a^ significant interaction by friend contact in lone-living adults (p=0.007)

# Supplementary Table 5 Characteristics of women and men in the EPIC-Norfolk cohort & sample of over-50s

|  | **Full cohort** | |  | **Over-50s** | |
| --- | --- | --- | --- | --- | --- |
|  | **Women** | **Men** |  | **Women** | **Men** |
| ***1993-1997*** |  |  |  |  |  |
| Age (yrs) at entry, mean (SD) | 59 (9.3) | 60 (9.3) |  | 62 (7.1) | 63 (7.1) |
| Female (%) | 55% | ̶ |  | 55% | ̶ |
| A-level or degree educated (%) | 46% | 61% |  | 48% | 62% |
| Social class I & II^a^ (%) | 42% | 46% |  | 44% | 49% |
| Deprived^b^ (%) | 17% | 16% |  | 15% | 13% |
| ***1996-2000 (HLEQ)*** |  |  |  |  |  |
| Married^c^ (%) | 74% | 88% |  | 74% | 89% |
| Living alone^c^ (%) | 21% | 12% |  | 23% | 12% |
| Rare friend contact^c^ (%) | 8% | 13% |  | 6% | 12% |
| Rare family contact^c^ (%) | 5% | 9% |  | 4% | 8% |
| ***1998-2002 (2HC)*** |  |  |  |  |  |
| BMI, mean (SD) | 26.5 (4.4) | 26.9 (3.3) |  | 26.7 (4.4) | 26.8 (3.3) |
| Excellent or good self-reported health (%) | 81% | 83% |  | 82% | 85% |
| Ever smoker (%) | 41% | 65% |  | 40% | 66% |
| Total energy intake (Kcal) | 1840 (518) | 2100 (596) |  | 1850 (516) | 2087 (583) |
| Score of Fruit variety, mean (SD) | 7.7 (2.3) | 6.7 (2.6) |  | 7.7 (2.3) | 6.7 (2.5) |
| Score of Vegetable variety, mean (SD) | 16.7 (4.0) | 16.0 (4.1) |  | 16.5 (4.0) | 15.9 (4.1) |

^a^ Social class I=professional, and class II=managerial and technical, occupations. ^b^ Area-level deprivation was based on the Townsend Deprivation Index (range: -6.1—6.98) with participants above average (>0.0) classified as deprived. ^c^ Measurement based on the postal Health and Life Experiences Questionnaire (HLEQ) (1996-2000); 2HC, second health check (1998-2002). Numbers in full cohort were: 25,639 at entry; between 7,883 and 11,232 at HLEQ; and between 15,000 and 17,165 at 2HC. Number of over-50s eligible for analysis was 9,580.
